# Recent Advances in Polymer-Inorganic Mixed Matrix Membranes for CO_2_ Separation

**DOI:** 10.3390/polym13152539

**Published:** 2021-07-31

**Authors:** Sipei Li, Yang Liu, Dana A. Wong, John Yang

**Affiliations:** Aramco Americas—Boston Research Center, Cambridge, MA 02139, USA; yang.liu@aramcoamericas.com (Y.L.); DAW710@ASCActiveDirectory.onmicrosoft.com (D.A.W.)

**Keywords:** polymer inorganic hybrids, mixed matrix membranes, CO_2_ separation

## Abstract

Since the second industrial revolution, the use of fossil fuels has been powering the advance of human society. However, the surge in carbon dioxide (CO_2_) emissions has raised unsettling concerns about global warming and its consequences. Membrane separation technologies have emerged as one of the major carbon reduction approaches because they are less energy-intensive and more environmentally friendly compared to other separation techniques. Compared to pure polymeric membranes, mixed matrix membranes (MMMs) that encompass both a polymeric matrix and molecular sieving fillers have received tremendous attention, as they have the potential to combine the advantages of both polymers and molecular sieves, while cancelling out each other’s drawbacks. In this review, we will discuss recent advances in the development of MMMs for CO_2_ separation. We will discuss general mechanisms of CO_2_ separation in an MMM, and then compare the performances of MMMs that are based on zeolite, MOF, metal oxide nanoparticles and nanocarbons, with an emphasis on the materials’ preparation methods and their chemistries. As the field is advancing fast, we will particularly focus on examples from the last 5 years, in order to provide the most up-to-date overview in this area.

## 1. Introduction

Environmental and energy issues are two of the major challenges mankind are facing in the 21st century. In the past few decades, as the concentration of CO_2_ in the atmosphere has continued to rise, the consequential effects of “global warming” and ecological destruction are threatening all living creatures on the Earth [1]. In 2021, the CO_2_ concentration in the atmosphere reached a record high, approaching 420 ppm, and it is expected to continue to rise in the near future. As a result, it has been a global endeavor to limit CO_2_ emission. In 2016, over 200 countries joined the United Nations to sign the Paris Agreement, indicating a consensus across the world about the critical need to reduce CO_2_ emissions [2].

In general, there are three major CO_2_ separation systems: (1) CO_2_/N_2_ separation in flue gas stream, (2) CO_2_/H_2_ separation in syngas, and (3) CO_2_/CH_4_ separation in natural gas production [3,4]. In addition to those, the direct air capture of CO_2_ from the atmosphere has also received a great amount of attention from both academia and industry [5]. Common CO_2_ separation techniques include cryogenic separation, absorption, adsorption, and membrane separation [6]. Cryogenic separation is based on the condensation of gases with different boiling points, and involves phase change, which also entails a high consumption of energy and thus may not be suitable for large-scale deployment. Chemical absorption is another widely commercialized separation technique, which relies on using a basic solvent to neutralize the acidity of CO_2_ by forming a weakly bonded intermediate. The absorption solvent can be further regenerated though heating (>100 °C). However, the absorption approach has several drawbacks, including equipment corrosion and degradation, high energy consumption, and potential environmental pollution [7]. Adsorption involves the physical capture of a gas or liquid on the surface of solid porous adsorbents. The adsorbents can be reversibly regenerated though two processes—temperature swing adsorption (TSA) and pressure swing adsorption (PSA). For TSA, the gas is attached to the adsorbent at high pressure and regenerated at low pressure. For PSA, the gas is attached to the adsorbent at low temperature and regenerated at an elevated temperature. However, both TSA and PSA also require significant amounts of energy and time to regenerate the adsorbents [8].

Membrane separation involves separating multiple types of gas molecules by taking advantage of the differences in permeability within the membrane under a given pressure. Although the commercialization of membrane separation only started in the 1970s, it has been almost two centuries since the discovery of the phenomenon of membrane separation. In 1829, Graham discovered the swelling of pig bladders in CO_2_ [9]. In 1831, Mitchell confirmed the difference between the diffusivity rates of H_2_ and CO_2_ in natural rubber [10]. In 1866, Graham proposed the solution–diffusion model, which explains the permeation of gas in polymers [11]. In the 1920s, Daynes invented a time lag method to measure the permeability coefficient [12]. In the 1930s, Barrer proposed dual mode sorption for gas permeation in glassy polymers [13]. Finally, in 1979, Monsanto commercialized a PDMS/PSf-based hollow fiber membrane under the name Prism^®^, with high permeability and selectivity, for several applications, including N_2_/O_2_ separation, gas dehydration, bio gas upgrading, etc. In comparison to the other CO_2_ separation techniques, the membrane separation technique has the advantages of easy processing, low cost and low energy consumption. Recently, the area of membrane separation has attracted much attention.

**Figure 1:**
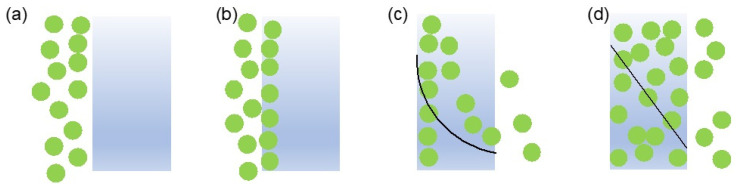
Graphic illustration of solution–diffusion model. (**a**) Upstream gas in contact with the membrane. (**b**) Dissolution of gas molecules into the surface of the membrane. (**c**) Diffusion of gas molecules through the membrane due to concentration gradient. (**d**) Gas molecules reached diffusion equilibrium with a linear concentration gradient through the membrane.

According to the structures of the membranes, they can be classified into two categories—nonporous (dense) membranes and porous membranes [14]. The solution–diffusion model is used to describe the transport behavior of vapor and gas through a nonporous membrane, by which it is assumed that no pores exist, and different gas molecules are separated based on their solubility and diffusivity inside the membranes [15]. In a solution–diffusion process, first, the upstream gas from the inlet (Figure 1a) come into contact with and dissolves in the high-pressure face membrane (Figure 1b). Then, the gas molecules diffuse through the membrane down a concentration gradient and desorb from the low-pressure face of the membrane (Figure 1c), where the diffusion is in a nonequilibrium state. Finally, as time goes on, eventually, the diffusion of gas reaches an equilibrium state (Figure 1d), and the one-dimensional gas flux (*N_A_*) across the membrane displays a linearity that can be described by Fick’s law [16]:

(1) $$N_{A}=-D_{A}\frac{dC_{A}}{dx}$$

where *D_A_* is the diffusion coefficient of gas *A* inside the nonporous membrane, *C_A_* is the concentration of molecule *A* inside the membrane, and *x* is the thickness of the membrane, ranging from 0 to approximately *l*.

Under the equilibrium state, the permeability of gas *A* across the membrane can be described as:

(2) $$P_{A}=\frac{N_{A}l}{p_{2}-p_{1}}$$

where *P_A_* is the permeability of gas *A*, normally expressed in the unit of Barrer (1 Barrer = 1 × 10^−10^ cm^3^(STP)cm/(cm^2^s cmHg)), *p*_2_ is the gas pressure on the upstream side (feed), and *p*_1_ is the gas pressure on the downstream side (permeate). According to Henry’s law, the gas concentrations at the high-pressure face (upstream) and the low-pressure face (downstream) are *C_A_*_1_ and *C_A_*_2_, and can be described as:

(3) $$C_{Ai}=S_{A}p_{Ai}\;\;\;(i=1,2)$$

where *S_A_* is the solubility of gas *A* in the membrane in an equilibrium state. Therefore, the permeability of gas *A* can be expressed as:

(4) $$P_{A}=D_{A}\times S_{A}$$

When there are two types of gases—*A* and *B*—the selectivity of gas *A* over gas *B* can be expressed as:

(5) $$\alpha_{A/B}=\frac{P_{A}}{P_{B}}=\frac{D_{A}}{D_{B}}\times \frac{S_{A}}{S_{B}}$$

For glassy polymers, the major contribution to selectivity is made by the ratio of the diffusion coefficient, which is dependent on the molecular sizes of the penetrant. For rubbery polymers, the major contributor to the selectivity is the ratio of solubility (sorption coefficient), which is dependent on the condensability of the gases in the membranes, as well as the gas–polymer intermolecular interactions.

For highly condensable gases, the concentration of the gas inside the membrane can be described by the Flory–Huggins model:

(6) $$\ln\frac{p}{p_{sat}}=\ln\phi_{2}+\;\left(1-\phi_{2}\right)\;+\;\chi {\left(1-\phi_{2}\right)}^{2}$$

where *p_sat_* is the saturated vapor pressure under test temperature, χ is the Flurry–Huggins coefficient, and $\phi_{2}$ is the volume fraction of gas dissolved inside the polymer.

As the temperature changes, the solubility of light gases remains relatively stable, while the solubility of highly condensable gases undergoes greater fluctuation. The relationship between gas *A*’s solubility inside the polymer and the temperature can be generalized as:

(7) $$S_{A}=S_{A0}\exp\;\left(-\frac{\Delta H_{S}}{RT}\right)$$

and the diffusivity of the gas can be generalized as:

(8) $$D_{A}=D_{A0}\exp\;\left(-\frac{E_{D}}{RT}\right)$$

where both *S_A_*_0_ and *D_A_*_0_ are constants, $\Delta H_{S}$ is the adsorption enthalpy, and *E_D_* is the activation of gas diffusion [16].

On the other hand, porous inorganic membranes have continued to receive attention from both academia and industry due to their well-known thermal and chemical stability, and their much higher gas flux and/or selectivity. When penetrant gases pass through a porous membrane, the gas flux is mainly determined by the pore sizes, and the selectivity hinges on the difference between the kinetic diameters of the gases and the pore size. In general, the separation mechanism can be described by the four following mechanisms: (1) Knudsen diffusion—when the upstream pressure is much higher than the downstream pressure, the selectivity is the square root of the ratio of the molecular weights of the penetrants; (2) capillary condensation—when the mean free path of the gases is between the smallest and largest pore sizes, the gases can be treated as a viscous fluid in terms of their permeability, and this is also determined by Knudsen diffusion; (3) surface diffusion—the driving force of gas diffusion is the concentration gradient of the gas near the wall of the pores; (4) molecular sieving—the porous membranes will block gases with a kinetic diameter greater than the pore size, and will only allow the passing of a gas with a smaller kinetic diameter [14,17].

**Figure 2:**
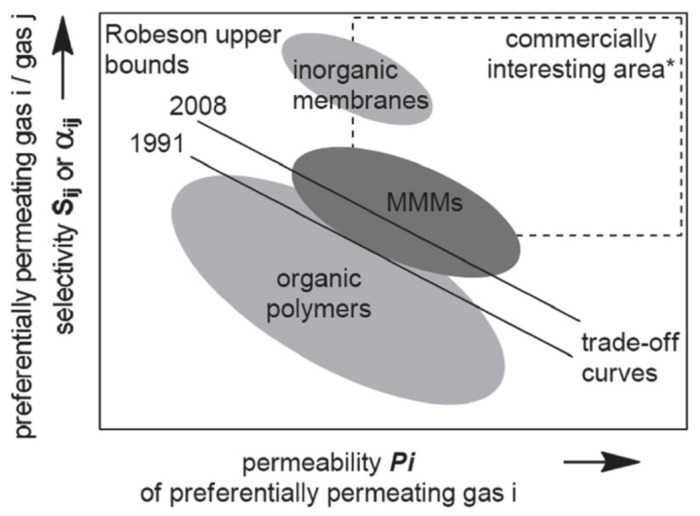
Schematic representation of the Robeson upper bounds (trade-off curves) from 1991 and 2008. Reprinted with permission from [20], copyright 2017 Wiley.

A typical composite membrane for gas separation is multilayered, whereby, on top of a microporous support layer (for mechanical stability), there are three layers of coating—a protective layer, a selective polymer layer and a gutter layer [18]. Commonly used porous support layers include polysulfone (PSf), polyacrylonitrile (PAN), cellulose acetate (CA), poletherimide (PEI), polyethersulfone (PES), polyvinylidene fluoride (PVDF), polyphenylene oxide (PPO), polypropylene (PP), polytetrafluoroethylene (PTFE), Teflon, etc. [19]. Both the gutter layer and the protective layer are highly permeable, and the desired gas selectivity is related to the selective polymer layer, which is usually 0.1 to 1 micron in thickness. In this paper, we will focus solely on the discussion of the selective polymer layer, especially in the context of the last 5 years. An ideal selective polymer layer should possess both high selectivity and permeability. However, membrane materials generally make a trade-off, with either high selectively/low permeability or vice versa. This phenomenon is exemplified by the Robeson-type trade-off plot, as shown in Figure 2, wherein the selectivity of gas pairs is logarithmically plotted against the permeability of the faster permeating gas. Tremendous effort has been made to create membrane materials that can exceed the Robeson limitation, as indicated in Figure 2.

Pure polymer membranes are particularly susceptible to the permeability–selectivity trade-off relationship, as depicted by the Robeson upper bounds [21]. A higher permeability with high selectivity allows for the use of a less polymeric material, ultimately reducing the cost, while a high selectivity results in the higher purity of the treated gas. Depending on the specific application case, a suitable combination of permeability/selectivity needs to be applied. In general, a commercially useful membrane should be in the upper right area of Figure 2. Many inorganic membranes, such as zeolite, have already reached this area. However, inorganic membranes are often compromised by brittleness and their difficult processing. Considering the nature of both polymer membranes and inorganic membranes, mixed matrix membranes (MMMs) [22], which consist of a polymer matrix as the continuous phase and a porous inorganic material dispersed in the polymer matrix as a filler, are becoming particularly appealing, as they combine the advantages of flexibility and high permeability, while canceling out the disadvantages of the two types of membranes, such as the trade-off of selectivity and permeability in polymer membranes and the brittleness in inorganic membranes.

Depending on the nature (porosity) of the inorganic fillers, there are two types of MMMs. When the inorganic filler is porous, the resulting MMMs can have drastically improved selectivity against smaller gases through the size exclusion effect [23], or against easily condensable gases by increasing the affinity of their inorganic nanopores towards the gas molecules [24]. If the inorganic filler is nonporous, such as nonporous oxide nanoparticles, the membrane’s free volume can be increased, especially for semi-crystalline polymers, such that the permeability of the membrane can be increased [25]. For MMMs with low inorganic loading, the effective permeability (*P_eff_*) can be described by the Maxwell model:

(9) $$P_{eff}=P_{c}\times \frac{P_{d}+2P_{c}-2\phi_{d}\times \;\left(P_{c}-P_{d}\right)}{P_{d}+2P_{c}+\phi_{d}\times \;\left(P_{c}-P_{d}\right)}$$

where *P_c_* is the permeability of the polymer (continuous) phase, *P_d_* is the permeability of the inorganic (dispersed) phase, and $\phi_{d}$ is the volume fraction of the inorganic phase, which can be calculated using the following equation:

(10) $$\phi_{d}=\frac{w_{d}/\rho_{d}}{\frac{w_{c}}{\rho_{c}}+\frac{w_{d}}{\rho_{d}}}$$

where $\rho_{c}$ is the density of the polymer phase and $\rho_{d}$ is the density of the inorganic phase. $w_{c}$ and $w_{d}$ are the weight fraction of the polymer and the inorganic filler. For porous fillers whose permeability is significantly higher than that of the polymer phase (*P_d_* ≫ *P_c_*), the effective permeability can be simplified as:

(11) $$P_{eff}=P_{c}\times \frac{1+2\phi_{d}}{1-\phi_{d}}$$

For nonporous fillers whose permeability is significantly lower than that of the polymer phase (*P_d_* ≪ *P_c_*), the permeability can be simplified as

(12) $$P_{eff}=P_{c}\times \frac{2-2\phi_{d}}{2+\phi_{d}}$$

However, when the inorganic loading exceeds a certain threshold (normally ~30 vol%), interconnection between the inorganic filler particles starts to occur. Under such circumstances, the MMMs act similarly to microporous membranes, with their inorganics connecting to form nanochannels [26]. Not only can the Maxwell model be used to estimate/predict the permeability of the MMMs with known *P_d_*, *P_c_* and $\phi_{d}$ (to help in understanding the structure–property relationship) [17], it can also be used to study the permeability and diffusivity of the inorganic fillers, which may be highly valuable when developing effective inorganic fillers. For example, Liu et al. developed an MMM based on 6FDA-DAM polyimide and a submicron-sized rare-earth (RE) MOF with a face-centered cubic (fcu) topology (RE-fcu-MOF). Such an MMM demonstrated exceptionally enhanced separation performance for the removal of CO_2_ and H_2_S from natural gas and the separation of butane isomers. In order to measure the permeability of different gases in the inorganic filler, the *P_eff_* of the MMM and the *P_c_* of the pure polymer phase were first experimentally measured. With known $\phi_{d}$, the *P_d_* values of RE-fcu-MOF were calculated. Furthermore, with the solubility of gas in the MOF filler obtained via the measured sorption isotherm, gas diffusivities in RE-fcu-MOF, including for He, H_2_, CO_2_, O_2_, N_2_, CH_4_, C_3_H_6_, n-C_4_, i-C_4_ and SF_6_, can be obtained [27,28,29].

## 2. Choice of Polymers and Inorganics

**Figure 3:**
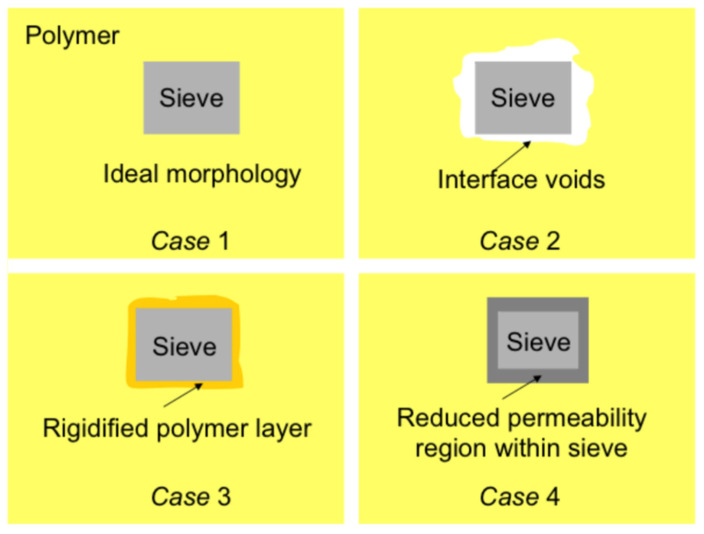
Four scenarios of mixing a polymer with inorganic fillers. Reprinted with permission from [17], copyright 2007 Elsevier.

The appropriate combination of polymer phase and inorganic phase, as well as preparation methods, are key to the performance of the resulting MMM. In general, there are four potential scenarios when mixing a polymer with inorganic fillers. Ideally, when the fillers are embedded in the polymer matrix without generating extra free volume and interaction with the polymer chains, the performance of the MMMs depends solely on the respective properties of the two phases, and can be described by the Maxwell model (Case 1 in Figure 3). However, especially for glassy polymers, the addition of fillers can generate extra free volume, as shown in case 2 of Figure 3, due to poor interfacial adhesion. As gases always prefer to move through the space of least resistance, permeability can increase at the cost of selectivity, as a result of “leaky interface”. Moreover, sometimes the addition of inorganic fillers can cause the rigidification of the surrounding polymers, thus decreasing the permeability with a non-significant increase in selectivity, as indicated in case 3. On the other hand, the polymer chains can either partially or completely block the pores in the inorganic fillers. Partially blocked pores may have a size exclusion effect, while completely blocked pores can turn the porous filler into nonporous filler [17,30].

CO_2_/N_2_ separation is often required for the treatment of flue gas from fossil fuel-fired power plants. Depending on the fuel source, flue gas often contains hot impurities, in addition to CO_2_ (10–15%) and N_2_ (78–80%), such as O_2_, CO, H_2_O, SO_2_, NO_x_, HCl, and Hg complexes, as well as considerable amounts of particles [31]. CO_2_/CH_4_ separation is employed mostly in the production of natural gas, the consumption of which has been increasing every year. Depending on the reservoir sources, raw natural gas contains various amount of CO_2_, hydrocarbons, H_2_S, N_2_, He, etc. [32]. Interest in CO_2_/H_2_ separation is relatively sparse compared to CO_2_/N_2_ and CO_2_/CH_4_ separation. The production of H_2_ is achieved through steam methane reforming (SMR), followed by water–gas shift (WGS), which leads to the combination of H_2_ and significant amounts of CO_2_ [33]. There are two types of CO_2_/H_2_ separation membranes—CO_2_-selective membrane, which rely on the difference of solubility in the membrane, and H_2_-selective membranes, which rely on the diffusivity of the gases in the membrane [19]. In order to meet the requirement of CO_2_ separation in different scenarios, the choice of polymer phase needs to satisfy several criteria, including (1) high gas permeance or permeability to reduce the membrane area; (2) high selectivity of CO_2_ over other gases so that the amount of gas to be circulated can be reduced; (3) high thermal and chemical stability due to the often harsh environment in which it will be used, such as high temperatures and corrosive acid impurities; (4) resistance to CO_2_ plasticization and aging to prevent the worsening of the separation efficiency over the long term; (5) low-cost and easy manufacturing for large-scale production [19].

**Scheme 1:**
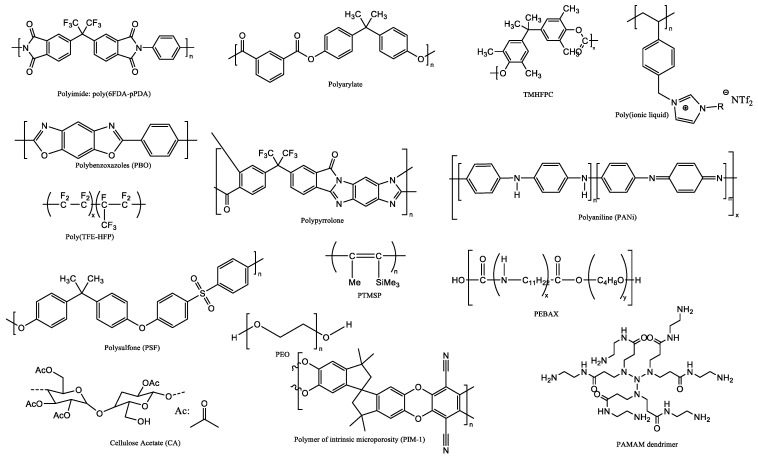
Typical polymer examples that are used for CO_2_ separation.

The commonly used polymers for CO_2_ separation include (Scheme 1):

(1) Polyarylates (PAs), which are synthesized through polycondensation between diol and dicarboxylic acid or dichloride [34]; (2) polycarbonates (PCs), which are synthesized via polycondensation between diol and phosgene, such as tetramethyl hexafluoro bisphenol-A polycarbonate (TMHFPC) [35]; (3) polyimides (PI) or polyetherimides (PEIs), which are synthesized through polycondensation between diamine and dianhydride under an aprotic polar solvent, usually using 4,4′-(Hexafluoroisopropylidene)diphthalic anhydride (6FDA), pyromellitic dianhydride (PMDA) or 3,3′4,4′-Benzophenonetetracarboxylic dianhydride (BTDA) as the dianhydride [36]; (4) polypyrrolones, which are similar to PIs but with considerably higher chain rigidity, and which are synthesized by polycondensation between dianhydride- and tetraamine-functionalized monomers [37]; (5) polysulfones (PSfs), which are usually synthesized by condensation between bisphenol A and dihalogenated diphenylsulfone [38]; (6) polyaniline (PANi), which is synthesized by oxidation polymerization [39]; (7) substituted polyacetylene, such as poly(1-trimethylsilyl-1-propyne) (PTMSP), with high permeability, which is synthesized by the polymerization of 1-trimethyl-silyl-1-propyne in the presence of TaCl_5_ and NbCl_5_ as catalysts [40,41]; (8) polyethylene oxide (PEO) [42] and its block copolymers [43,44]; (9) polyether block amides (PEBA) under the trade name of Pebax, which are synthesized by the polycondensation of a carboxylic acid polyamide (PA6, PA11, PA12) with a hydroxyl-terminated polytetramethylene glycol (PTMG) [45]; (10) poly(amidoamine) (PAMAM) dendrimers, which are synthesized by two-step addition polymerizations with a high abundance of terminal amine groups [46]; (11) cellulose acetates (CAs), which are produced via the acetylation of hydroxyl groups of cellulose, leading to reduced chain packing and high CO_2_ permeability [32,47]; (12) perfluoropolymers, such as poly(tetrafluoroethylene) (PTFE) or poly (hexafluoropropylene–co-tetrafluoroethylene) (poly(HFP-TFE)), which have high chemical and thermal stability against plasticization and aging, as well as high processability due to their low viscosity in fluorinated solvent [48]; (13) polybenzoxazoles (PBOs), which have both high permeability and selectivity while being insoluble in most solvent, thus providing security against membrane plasticization [49]; (14) poly(ionic) liquids (PILs), which are ionic liquids immobilized on a polymer backbone and which have high solubility in CO_2_ [50]; (15) polymers of intrinsic microporosity (PIMs), which have a high degree of inter-chain separation and rigidity, leading to internal microporosity that can separate CO_2_ with both high selectivity and permeability [51].

There are several major types of inorganic fillers that have been widely used to prepare MMMs: (1) Zeolite—a class of microporous, aluminosilicate minerals commonly used as commercial adsorbents for separation and as catalysts due to their high surface area and chemical stability [52,53]; (2) metal organic framework (MOF)—a class of polymers composed of metal ions or clusters coordinating as organic ligands to form highly porous structures with a high surface area, low density and structural tunability [54,55]; (3) zeolite imidazolate framework (ZIF)—a subclass of MOF materials, composed of metal ions connected by imidazole with tetrahedral structures, that have flexible structures and adjustable pore sizes and better thermal, chemical and moisture stability than other types of MOFs [56]; (4) oxide nanoparticles, such as non-porous silica or metal oxide nanoparticles, mesoporous silica, polyoligosilsesquioxanes (POSS), etc. [57,58,59,60]; (5) nanocarbons such as 0-D carbon quantum dots, 1-D carbon nanotubes (CNT), 2-D graphene, 3-D m carbon molecular sieves (CMS), activated carbon (AC), etc. [61,62,63,64].

## 3. Polymer/Zeolite Mixed Matrix Membranes

Table 1 listed recent examples of zeolite based MMMs for CO_2_ separation. Asghari et al. prepared a PEBAX/zeolite MMM with 1 wt.% loading of Zeolite 13X deposited on a PSf/PE substrate layer. The CO_2_ permeability and CO_2_/N_2_ and CO_2_/CH_4_ selectivity for the MMM loaded with 1 wt.% zeolite 13X showed increases of 200% (194.12 Barrer), 1030% (56.5) and 3633% (56) compared to the neat membrane [65]. Jusoh et al. reported an MMM with 1 wt.% zeolite T embedded in 6FDA-durene polyimide. The composite membrane showed a CO_2_ permeability of 843.6 Barrer and an ideal CO_2_/CH_4_ selectivity of 19.1, which values are 80% and 172% higher than those of the neat membrane, along with enhanced resistance to plasticization up to 20 Bar [66]. Ahmad et al. studied the effect of adding 25 wt.% zeolite 4A to polyvinyl acetate (PVAc), and found that the addition of zeolite 4A increased the CO_2_/N_2_ selectivity by 75% (100.54), although with a reduction in permeance [67]. Adams et al. studied the high loading of zeolite 4A (up to 50 vol.%) onto PVAc, and found that even with such high inorganic loading and at high pressure (440 psi), an improved selectivity (63%) for CO_2_/CH_4_ could be observed [68]. Zhao et al. reported a type of MMM based on Matrimid^®^ 5218 polyimide and a partially lithiated Na-ZSM-25 zeolite (LNZ-25). Due to the complete blocking of CH_4_ and the high admission of CO_2_ achieved by LNZ-25, the MMM with 5 wt.% zeolite loading showed a high CO_2_/CH_4_ selectivity of 169 at 5 bar, as well as plasticization resistance behavior [69]. Zhang et al. reported a tertiary-component MMM based on doping Pebax polymer with PEG-600 and NaY zeolite. The MMMs with 20 wt.% PEG and 30 wt.% NaY zeolite showed CO_2_/N_2_ selectivity of 107.9 and CO_2_ permeability of 172.6 Barrer. Furthermore, with the CO_2_/N_2_ mixed gas, the designed membrane could directly enrich the concentration of CO_2_ from 15% to 96.7% in one step [70].

Li et al. recently reported a carbon molecular sieve-type membrane based on a combination of hierarchically porous zeolite 5A and a thermally carbonized Matrimid^®^ polyimide polymer. The hierarchical zeolite 5A with both micropores and mesopores can facilitate CO_2_ diffusion and reduce transport resistance at the same time; the thermal carbonization further improved free volume and pore aperture. This carbon molecular sieve-type MMM showed 450 Barrer of CO_2_ permeability and 19.3 Barrer of CO_2_/CH_4_ selectivity [71].

**Figure 4:**
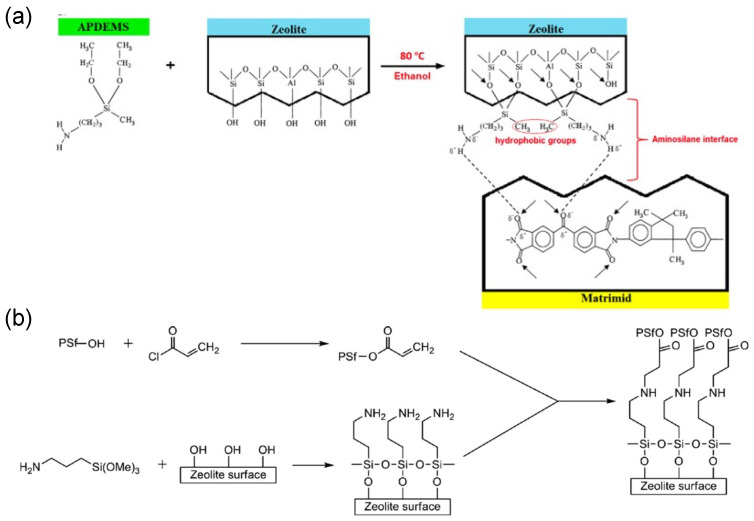
(**a**) The grafting reaction between APDEMS and the zeolite surface. Reprinted with permission from [74], copyright 2015 Elsevier. (**b**) Reaction scheme of covalently bonded zeolite with acrylate modified polymer. Reprinted with permission from [75], copyright 2010 Elsevier.

In order to enhance the compatibility between the zeolite and the polymer matrix, zeolite modification or the adding of a third phase agent were often employed. Mashhadikahn et al. functionalized the zeolite 13X with aminosilane, which reacted with the hydroxyl groups on the zeolite particles. The functionalized zeolite particles, with an average pore size of 2.57 nm, were mixed with 6FDA-durene polyimide and fabricated into a membrane though solution-casting. With 15 wt.% loading of the aminosilanized zeolite, the polyimide-based MMM showed a high CO_2_ permeability of 887 Barrer and a CO_2_/N_2_ selectivity of 25.3 Barrer at a feed pressure of 0.2 MPa at room temperature [72]. Similarly, Chen et al. modified EMC-2 zeolite with 3-aminopropyltriethoxysilane (APTES) as the amino agent and APTMDS (bis(3-aminopropyl)-tetramethyldisiloxane) as the crosslinker [64]. The crosslinked MMM with 6FDA-ODA at 25 wt.% zeolite showed excellent CO_2_/CH_4_ separation at 150 psi and 35 °C, with a CO_2_ permeability of 40.9 Barrer and a CO_2_/CH_4_ selectivity of 80.2 Barrer [73]. Amooghin et al. created defect-free Matrimid^®^ 5218/NaY zeolite by using 3-aminopropyl (diethoxy)methylsilane (APDEMS) as the crosslinking agent (Figure 4a) [65]. The obtained results revealed that at 15 wt.% of filler loading, the CO_2_ permeability increased from 8.34 Barrer for the neat membrane to 9.70 Barrer, and the CO_2_/CH_4_ selectivity was considerably increased from 36.3 to 57.1 Barrer (about 57%) due to the improved dispersion of the inorganic fillers [74]. Khan et al. developed a coupling approach by using APTMS to crosslink zeolite 3A with arylate-terminated PSf, as shown in Figure 4b [66]. The crosslinked PSf/zeolite 3A MMM with 40 wt.% zeolite loading exhibited a H_2_ permeance of 15.1 GPU and H_2_/CO_2_ selectivity of 7.12 Barrer, which is an approximately 5-fold increase compared to the neat membrane [75]. A similar approach was used to prepare the crosslinked PSf/MCM-41 MMMs, which showed high ideal selectivities for CO_2_/N_2_ and CO_2_/CH_4_ of 32.97 and 31.48 Barrer, respectively [76].

In another approach to enhancing the compatibility between zeolite and the polymer matrix, Ahmad et al. treated polysulfone (PSf)/silicoaluminophosphate (SAPO)-34 zeolite MMM with 1-ethyl-3-methyl imidazolium bis(trifluoromethylsulfonyl)imide ([emim][Tf2N]) ionic liquid, in order to seal the interfacial defect. The CO_2_/N_2_ selectivity and CO_2_ permeance were increased to 44.9 and 7.19 GPU, respectively, with 5 wt.% of filler loading at 3.5 Bar and 30 °C [77]. Shindo et al. also studied the effect of adding ionic liquid ([C4mim][Tf2N]) to an MMM containing 6FDA-TeMPD polyimide and zeolite ZSM-5 [70]. It was evident that with the addition of IL, the interfacial voids were reduced, with a consequentially improved CO_2_ selectivity against both N_2_ and H_2_ [78]. Singh et al. reported an MMM containing poly(ionic) liquid (PIL) crosslinked with divinylbenzene, room temperature ionic liquid (RTIL) and SAPO-34 zeolite particles [71]. Such a system showed high solubility for CO_2_ and high mechanical stability, and thus a CO_2_/CH_4_ selectivity of 90 and CO_2_ permeability of 26 Barrer [79]. Dunn et al. evaluated the zeolite-PIL-RTIL MMM under a binary feed of up to 40 bar at 50 °C [68]. It was found that the SAPO-34-PIL-RTIL MMM with an inorganic loading of 30 wt.% showed a CO_2_/CH_4_ selectivity of 43, with a CO_2_ permeability of 202 Barrer, under an equimolar binary feed [80].

## 4. Polymer/MOF Mixed Matrix Membranes

**Figure 5:**
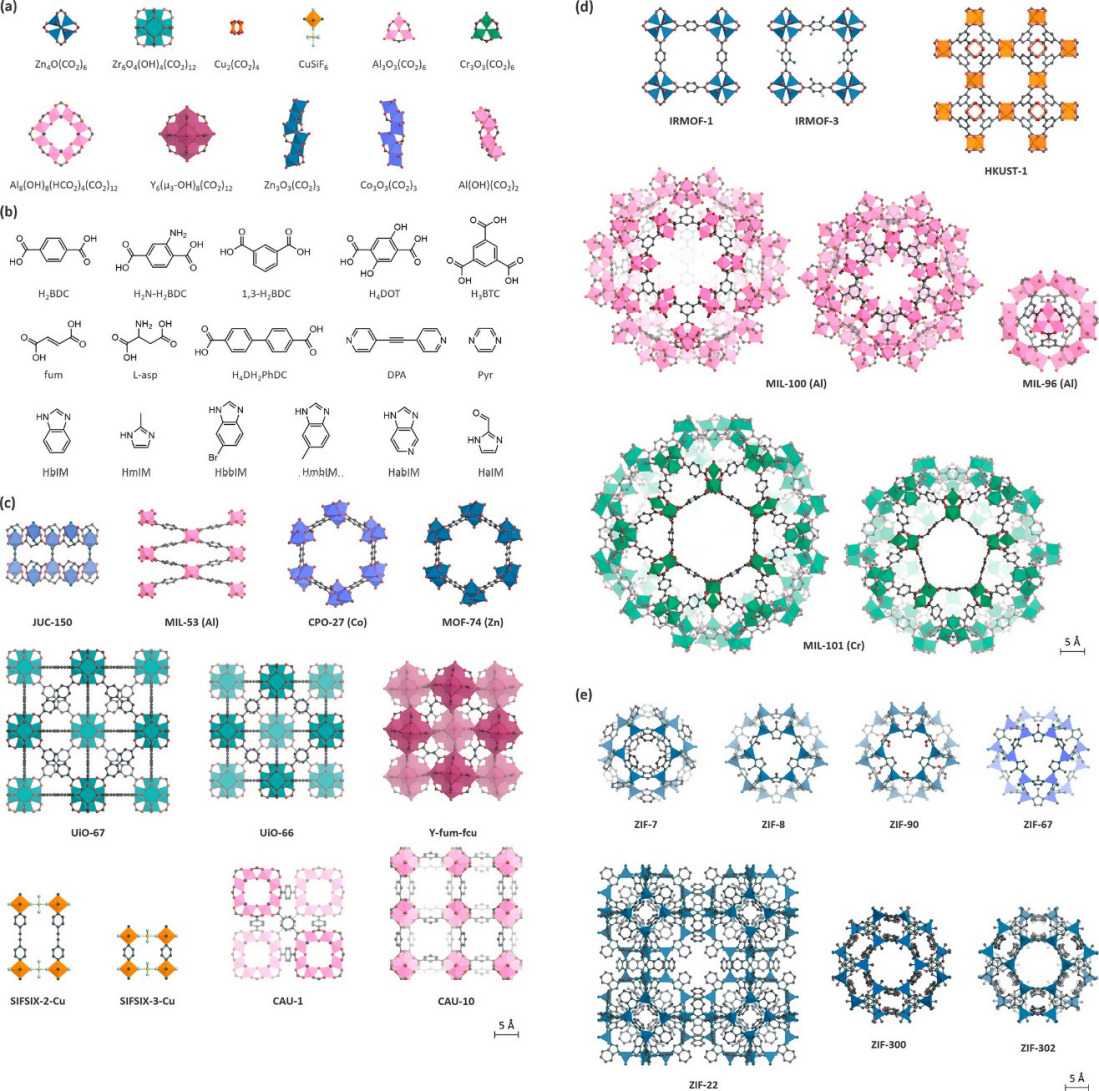
Representative MOFs for mixed matrix membrane developments. (**a**) Inorganic secondary building units (SBUs) and (**b**) organic linkers of MOFs. (**c**–**e**) MOF structures of particular interest to membrane-based gas separation applications. Reprinted with permission from [84], copyright 2020 ACS.

MOFs represent a class of highly ordered crystalline porous materials [81,82]. MOFs are considered promising molecular sieves for various separation applications due to their exceptionally high surface area, tunable pore/channel sizes, and tailorable functionalities [82]. MOF-based mixed matrix membranes (MOF-MMMs) are developed as a compromise to overcome these practical challenges in membrane fabrication and utilization [83]. Promisingly, thanks to the better chemical compatibility between MOFs and polymers, the interfacial issue is less severe in MOF-MMMs than in zeolite-MMMs [20,83]. In this regard, numerous MOF-MMMs have been reported in recent years. The remarkable progresses in, and remaining challenges for, MOF-MMMs have been extensively summarized in several insightful reviews [84,85,86,87]. In this miniature review, we mainly focus on the most commonly studied MOFs involved in the development of MOF-MMMs for CO_2_-related separation, and discuss several practical factors affecting membrane separation performance. Some examples of MOF structures are summarized in Figure 5.

Indeed, among the thousands of MOFs reported, only a small portion have been utilized as efficient fillers for CO_2_/H_2_, CO_2_/N_2_ and CO_2_/CH_4_ separations. This is probably due to the similar physical and chemical properties among the gas molecules, requiring the precise control of MOF structures to achieve optimal molecular sieving affinity. Nevertheless, in the past 5 years, x-UiO-66 (x = functional group), x-MIL-53, and ZIF-8 have received extensive interest as the three most studied MOF fillers for CO_2_-related separations, based on our analysis of accessible publication records, accounting for over half of the total. On the one hand, the former two kinds of MOFs, i.e., NH_2_-UiO-66 and NH_2_-MIL-53, capture CO_2_ from mixtures as a result of favorable CO_2_–host interaction affinities, which can be further rationally tuned by the highly amendable functionality (x) of the linkers. On the other hand, ZIF-8 separates gas molecules through its ideal pore aperture (~3.4 Å), allowing target molecules to pass through with relative ease, but hindering other molecules.

Table 2 listed some of the recent examples of MOF/polymer MMMs. Urban and co-workers examined high NH_2_-UiO-66 loadings (~50 wt.%) in polysulfone (PSf), and observed the evolution from single-mode to dual transport regimes [88]. A new percolative pathway though the MOF is formed, acting as a molecular highway for gases when the MOF loading increases above 30 wt.%. As a result, CO_2_ permeability dramatically increased from 18 to 46 Barrer, while the CO_2_/CH_4_ selectivity was well maintained (~24–26).

Zhu and co-workers functionalized the NH_2_-UiO-66 structure by converting the -NH_2_ into -CN groups, which covalently linked with the PIM-1 via thermal reaction. The UiO-66-CN@PIM-1 membranes showed exceptionally high CO_2_ permeability (>15,433 barrer) and high CO_2_/N_2_ selectivity (>24) [89].

Friess and coworkers incorporated NH_2_-UiO-66 into a polyethylenimine (PEI) matrix with an MOF loading of 18 wt.%, and observed a CO_2_ permeability of 394 Barrer with a CO_2_/H_2_ selectivity of ~12 [90]. Interestingly, Xiang and coworkers combined polyvinylpyrrolidone-modified PA-UiO-66 particles (PVP + MOF) with poly(acrylic acid) (PAA) through H-bonding-assisted layer-by-layer assembly, and achieved a H_2_/CO_2_ selectivity of ~20, indicating the highly amendable nature of mixed matrix membranes for separating the mixture of H_2_ and CO_2_ [91].

Chen and coworkers fabricated Pebax-based composite hollow fiber membranes by incorporating NH_2_-UiO-66 into the thin Pebax selective layer, and observed excellent CO_2_ permeance (~325 GPU) and CO_2_/N_2_ selectivity (~56), as well as CO_2_/CH_4_ selectivity (~20), with a loading of 50 wt.%. In addition, the authors found that the presence of UiO-66 in Pebax significantly improved the membrane’s operational stability under high pressure [92].

Rodrigue and coworkers fabricated MMMs using NH_2_-MIL-53(Al) particles as fillers and rubbery Pebax as the matrix, via a solution casting method [93]. The authors achieved enhanced CO_2_ permeability, as well as CO_2_/N_2_ and CO_2_/CH_4_ selectivity, by introducing the MOF. The highest ideal selectivity values for CO_2_/CH_4_ and CO2_2_/N_2_ separations are 23.3 and 59.4 Barrer, respectively, with 10 wt.% MOF loading, while the CO_2_ permeability was as high as 149 Barrer.

Fong and coworkers optimized the spinning parameters of NH_2_-MIL-53(Al)/CA hollow fiber MMMs for gas separations [94]. The authors concluded that the optimization of spinning parameters can be considered a feasible and efficient method to fabricate hollow-fiber MOF-MMMs with improved separation performance.

Li and coworkers tailored the interfacial interaction between ZIF-8 and the polymer matrix, and observed an exceptional CO_2_/CH_4_ selectivity of 58 Barrer with a CO_2_ permeability of 20 Barrer [95]. The authors revealed that the Zn^2+^ post-modification of the polymer matrix enables enhanced polymer/ZIF-8 interaction, while controlling the Zn^2+^ treatment conditions can further tailor the CO_2_ permeability and CO_2_/CH_4_ selectivity. Wey and coworkers used thermally annealed ZIF-8 nanoparticles as fillers in poly(styrene-co-butadiene) (SBC) to improve the permselectivity of MMMs [96]. The thermal annealing treatment created polar atoms by breaking the Zn–N bonds in the ZIF-8 framework, and these are beneficial in promoting both CO_2_ permeability and CO_2_/N_2_ selectivity. The fabricated MMMs with a ZIF-8 loading of 20 wt.% exhibit a CO_2_ permeability of ~40 Barrer, with a CO_2_/N_2_ selectivity of ~22 Barrer. More examples of ZIF-MMMs will be discussed in Section 5.

Several other MOFs have also been widely studied in MMM development. For instance, Guiver and coworkers constructed ultrathin unobstructed gas transport channels in MMMs by using the gravity-induced interface self-assembly of poly(vinylamine) and polymer-modified MIL-101(Cr) [97]. Promisingly, the MMMs achieved a high CO_2_ permeance of 283 GPU (gas permeation units), and a CO_2_/N_2_ selectivity of 242 Barrer at 0.5 MPa.

Koros and coworkers investigated the CO_2_/CH_4_ separation performance of a Y-fum-fcu-MOF/6FDA-DAM MMM over a wide range of temperatures from 233 K to 338 K [98]. The authors revealed that temperature can significantly affect the membrane performance due to the different roles that sorption and diffusion factors played in the separation process. Remarkably, decreasing the temperature from 308 K to 233 K affords an extremely high CO_2_/CH_2_ selectivity (~130), with an associated CO_2_ permeability above 1000 Barrer.

Besides the selection of MOF fillers and polymer matrices, the interfacial compatibility between the MOF and the polymer is another key consideration to realizing the full potential of MOF-MMMs for gas separation. Li and coworkers reported a new approach using polyimide brushes to covalently graft the MOF surface to the engineer interface, which resulted in a stand-alone membrane with 88 wt.% MOF loading [99]. The same group further reported a dual-interfacial engineering approach using a sub-20 nm polycrystalline MOF-74 shell as a transition phase to engineer the MOF–polymer interface [100]. Following this novel approach, the NH_2_-UiO-66-based MMMs manifested a simultaneous increase in CO_2_/CH_4_ selectivity and CO_2_ permeability with increases in MOF loading.

In summary, while there is as yet no commercialized MOF-based MMM, they still hold huge potential for the gas separation industry. Of the polymers adopted as the matrix, the commercial polymer Pebax has received major attention. In addition, 6FDA-based polyimide is another hot candidate for use as a polymer matrix. On the other hand, ZIF and UiO-66 are the most popular MOF materials.

## 5. Polymer/ZIF Mixed Matrix Membranes

**Figure 6:**
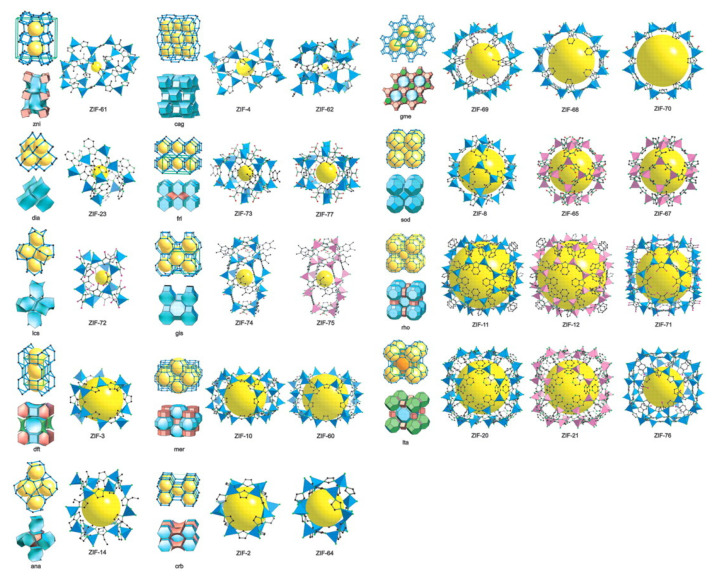
The crystals of ZIFs. The largest cage in each ZIF is shown with the ZnN_4_ tetrahedra in blue and the CoN_4_ in pink. The yellow ball is included in the structure for clarity and to indicate space in the cage. Reprinted with permission from [101], copyright 2008 American Association for the Advancement of Science.

ZIFs, a subfamily of MOFs, possess a zeolite topology with tunable pore structures. ZIFs consist of point metal nodes in a center connected to imidazolate linkers, and can adopt numerous three-dimensional structures (Figure 6) [101]. Before now, 15 ZIF types have been found among 239 known types, and the relationship between imidazolates and topologies has been described in detail in the literature [102].

ZIFs have gained technical and scientific attention due to their pore–structural network, and have many potential applications in the fields of photocatalysis [103], drug delivery [104], pharmaceutical compounds separation [105,106], dye-wasted water treatment [107], oil–water separation [108], water purification [109,110], the pervaporation recovery of solvent vapors [109,111,112,113], electrocatalysts [114], solvent-resistant nanofiltration [115], gas–liquid separation [116], natural gas separation [117,118,119] and so on.

As described in Figure 2, MMMs may provide separation properties that can overcome the Robeson upper bound. Different types of inorganic fillers including ZIFs have been reported in recent reviews [22,120,121,122]. Typical ZIFs such as ZIF-7, ZIF-8, ZIF-11, ZIF-67, ZIF-71, ZIF-90, ZIF-95, ZIF-301, ZIF 302, etc., have been fabricated as sole ZIF MMMs [123,124,125,126,127,128,129,130,131,132,133,134,135,136,137,138,139] or modified ZIF MMMs [95,126,140,141,142,143,144,145,146,147,148,149,150,151,152,153,154,155,156,157,158,159,160] for different gas separation applications, such as for CO_2_/CH_4_, CO_2_/N_2_, H_2_/CH_4_, C_3_H_6_/C_3_H_8_, etc. For example, Guan et al. highlighted recent research progress in ZIF-based MMMS for CO_2_ separation [118]. The synergistic effect of the modified ZIFs can change the structure of the membrane with enhanced interfacial compatibility, and thus improve the separation performance. The effects of the characteristics of these ZIFs, such as their filler size, dispersibility, and hydrophilic and hydrophobic nature, can affect membrane separation performance.

Some ZIF materials, such as ZIF-8, ZIF-11, ZIF-67, ZIF-71, ZIF-90, ZIF-95 and ZIF-301, have large cavities with narrow pore apertures close to the kinetic diameter of CO_2_ (0.33 nm), and this allows the inclusion of a suitable filler material in sole ZIF-based MMMs for CO_2_ separation. Table 3 compiles the research studies carried out in the past 5 years on the fabrication of sole ZIF-based MMMs.

Among the ZIF series, ZIF-8, with a diameter of ca. 1.1 nm and 0.34 nm windows, is frequently studied for MMM separation performance [125,126,128,129,130,131,132,133,136,139]. Two major challenges ((1) high ZIF-8 loading and (2) the fabrication of ultrathin (~100 nm) ZIF-8-based MMMs) need to be overcome in order to commercialize ZIF-8-based membranes [117]. ZIF-11 is a promising ZIF, with structural features (connected via apertures with a diameter of 0.3 nm) that make it appropriate for H_2_ or CO_2_ separation. Ehsani et al. utilized ZIF-11 as the filler and Pebax 2533 as the membrane matrix to fabricate ZIF-11/Pebax 2533 rubbery MMMs [137]. The resulting membranes exhibited the highest CO_2_ permeability (402.9 Barrer) and selectivity for CO_2_/CH_4_ (12.5), H_2_/N_2_ (12.9) and H_2_/CH_4_ (4.8) at 50-70 wt.% loading. ZIF-67 has also attracted considerable attention due to its natural family of MOFs. An et al. [139] first produced ZIF-67/6FDA-DAM MMMs for C_3_H_6_/C_3_H_8_ separation, and Meshkat et al. successfully incorporated ZIF-67 in a Pebax 1657 rubbery membrane to obtain an MMM [130]. Such a membrane not only improved gas permeability (130% increase in CO_2_ permeability), but also enhanced membrane separation performance (58% increase in CO_2_/CH_2_ selectivity), compared to a pristine Pebax membrane at a feed pressure of 11 bar and at 35 °C. ZIF-95 with large voids (24.0 Å) is also an extremely important material in gas adsorption and purification applications. In the work of Ilicak et al., ZIF-95 was used as filler in the preparation of polyimide-based MMMs [135]. ZIF-95/Matrimid 5218 (70/30) MMMs achieved good CO_2_ separation performance (CO_2_/CH_2_ selectivity of 58 and H_2_/CH_2_ selectivity of 192.0). These results indicate filler–polymer compatibility, and the possibility of forming additional channels and enhancing the free Matrimid volume.

In order to improve the interfacial compatibility between ZIFs and polymers in MMMs, and thus membrane separation performance, many methods of modifying and functionalizing ZIFs so as to build covalent bonds between ZIFs and polymers in the membrane matrix have been explored. Table 4 compiles the research studies carried out in the past 5 years on the fabrication of modified and/or hybrid ZIF-based MMMs for gas separation. The strategy of ZIF modification not only greatly broadens the scope of choice for polymer matrices and fillers; it also further enhances the membrane separation performance due to the extra functional groups introduced to overcome the problems of weak polymer–filler interfacial interactions [118].

ZIF-7 is another promising member of the ZIF family for CO_2_ separation. Gao et al. incorporated functionalized OH-ZIF-7 filler into a Pebax 2533 matrix [156]. The resulting OH-ZIF-7/Pebax MMMs showed high CO_2_ permeability of 273 Barrer and a CO_2_/N_2_ separation factor of 38. In the work of Wang et al., a thin and uniform polydopamin (PD) layer of well-controlled thickness was coated on the surface of ZIF-8 nanocrystals via a self-polymerization process of dopamine, in order to improve the interfacial interaction of ZIF-8 nanocrystals and polyimide with a Tröger’s base functional group (TBDA2-6FDA-PI) [124]. Such MMMs achieved excellent gas permeation performances and comprehensive gas separation performances, surpassing the Robeson upper bound for gas pairs of H_2_/N_2_ and H_2_/CH_4_, and approaching the upper bound for O_2_/N_2_ gas pairs. Several researchers fabricated robust MMMs using amino-functionalized ZIFs (e.g., NH_2_-ZIF-7 and NH_2_-ZIF-8) with rubbery polymers (e.g., XLPEG, Pebax) [142,158] and glassy polymers (e.g., 6FDA-PI, PSF, polyamide) [128,143,156]. These amino groups enhanced filler affinity for CO_2_, resulting in significantly improved separation performances (CO_2_/N_2_ and CO_2_/CH_4_ selectivities) in the resultant MMMs, compared to the neat polymer under the same testing conditions.

In order to improve gas molecule transportation in the membrane matrix between ZIFs and polymers, ZIF hybrids—a combination of ZIFs with other inorganic fillers (e.g., SWCNTs, MWCNTs, GO, SiO_2_, etc.)—were developed and used as fillers for gas separation [125,126,145,146,147,148,149,150,152,153,157,159]. Li et al. developed novel MMMs by incorporating ZIF-8, in situ-inserted by MWCNTs, into a Pebax 1657 membrane matrix [150]. The functionalized MWCNTs inserted into the ZIF-8 particles not only prevented the aggregation of ZIF-8 particles, but also enhanced the membrane permeation properties (CO_2_ permeability of 186 Barrer and CO_2_/N_2_ selectivity of 61.3 at feed pressure of 0.5 MPa and 35 °C) and membrane mechanical strength. Huang et al. prepared a ZIF-8@GO hybrid using a facile in situ growth method, and accordingly fabricated ZIF-8@GO/Matrimid MMMs [153]. Such MMMs containing 20 wt.% ZIF-8@Go hybrid exhibit the highest selectivity of up to 65 for CO_2_/N_2_, with a CO_2_ permeability of 238 Barrer, which surpasses the Robeson upper bound for 2008.

**Figure 7:**
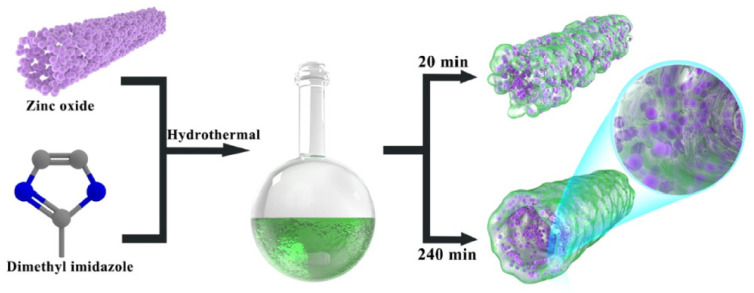
Synthesis process of ZIF-8 hollow nanotube. Reprinted with permission from [125], copyright 2021 Elsevier.

Most ZIFs are particle-like and have a 3D framework [101,102]. Recently, new kinds of ZIFs with other shapes have been prepared and used as fillers for the fabrication of MMMs. Wang et al. fabricated MMMs by dispersing ZIF-8 hollow nanotubes (H-ZIF-8) (Figure 7) in a Pebax 1657 membrane matrix [125]. Compared with neat Pebax, the MMMs containing 5% H-ZIF-8 achieved promising CO_2_ separation performances (CO_2_ permeability of 147 Barrer and CO_2_/N_2_ selectivity of 68), approaching the 2008 Robeson upper limit and surpassing most reported CNTs/Pebax-based membranes.

**Figure 8:**
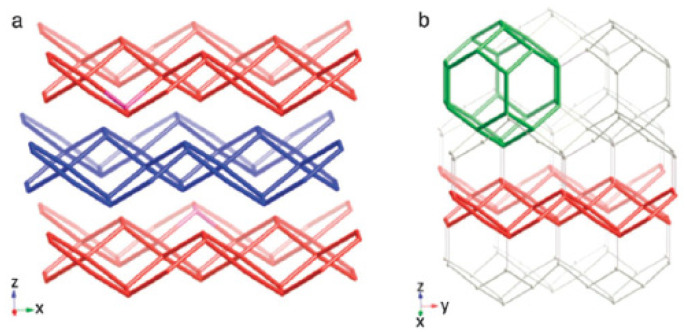
(**a**) 2-D ZIF-L along z, (**b**) sod topology of ZIF-8. Relationship between ZIF-8 and ZIF-L: ZIF-8 framework (grey color) with a sodalite cage highlighted in green, and the layer corresponding to ZIF-L in red. Reprinted with permission from [161], copyright 2013 RSC.

Recently, 2-D nanoporous nanosheets have attracted significant attention due to their beneficial features associated with lower diffusion resistance and large external surface area, the latter of which allows the exposure of more active sites. The 2-D ZIFs are a new kind of ZIF, with a cushion-shaped cavity between the layers that has dimensions of 0.94 nm × 0.7 nm × 0.53 nm and a leaf-shaped structure (named ZIF-L in Figure 8; reported and prepared by Chen et al.) [161]. Feng et al. used 2-D ZIF and 2-D ZIF-derived nanoporous nanosheets in various applications due to their special morphology and pore features [162], including metal removal [163], water purification [164], solvent separation [111], gas separation [165,166,167,168], and so on.

**Figure 9:**
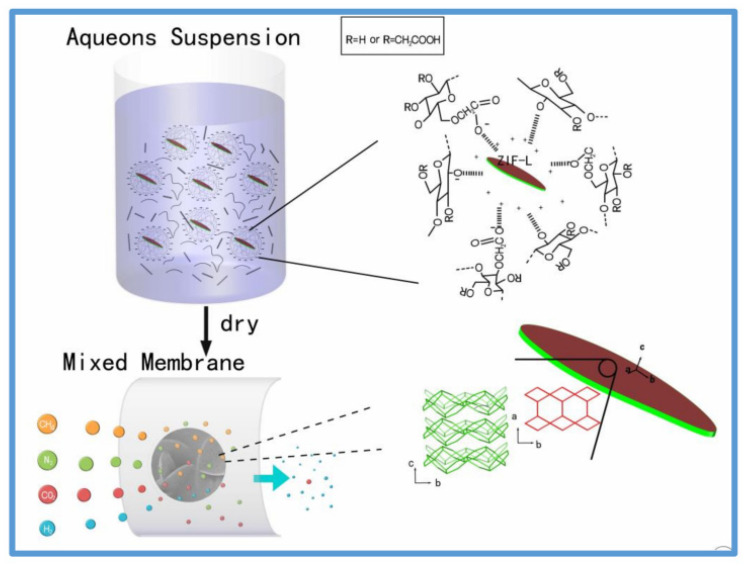
Fabrication of CMC-ZIF-L nanoporous composite membrane for gas separation applications [166].

In a recent work by Deng et al., the effect of the morphology of ZIF on the CO_2_ separation ability of ZIFs/Pebax 2533 MMMs was investigated [168]. Three ZIFs with different shapes—particles (0D), microneedles (1D) and leaves (2D)—were studied. The results show that the increment in CO_2_ permeability was related to the ZIF morphology, in the order of 0-D < 1-D < 2-D. The effects of morphology on selectivity seem to be the opposite, with the 0-D ZIF showing the highest selectivity. Zhang et al. synthesized 2-D ZIF-IL nanosheets using zinc salt and 2-methylimidazole aqueous solution, and incorporated these into a carboxymethyl cellulose (CMC) solution to form a steadily mixed aqueous suspension through one-step solution blending (Figure 9) [166]. The resulting MMMs with a 30 wt.% loading of ZIF-IL exhibited the highest separation ability (the selectivities of H_2_/CO_2_, H_2_/N_2_, CO_2_/CH_2_ and N_2_/CH_2_ were 10.62, 21.54, 17.87 and 8.93, respectively).

Zhu et al. proposed the functionalized auxiliary etching of ZIF-L fakes (hZIF-IL) by tannic acid (TA) to enlarge the surface or the pores, and enhance the hydrophilicity [165]. The as-prepared Pebax 1657/hZIF-IL MMM with 5 wt.% loading of hZIF-IL showed significant improvement in membrane transport properties (40% increase in CO_2_ permeability and 70% increase in CO_2_/CH_4_ selectivity), compared to the neat Pebax 1657 membrane. Kim et al. reported the preparation of PI-ZIF-IL MMMs by incorporating ZIF-IL nanosheets and OH-containing polyimide for H_2_ separation [167]. The transport properties of such MMMs with 20 wt.% ZIF-IL included H_2_ permeability of 260 Barrer and H_2_/CO_2_ selectivity of 13.4 at 1 bar feed pressure and 25 °C. This PI-ZIF-IL MMM achieved outstanding H_2_ and CO_2_ separation performance—exceeding the latest Robinson’s upper bound—compared to ZIF membranes and their MMMs with different ZIFs and polymer matrices.

In summary, Pebax, 6FDA-based polyimide and PSf are the best options for use as the polymer matrix for ZIF-based MMMs. ZIF-7 and ZIF-8, together with their functionalized counterparts, received the most attention due to their excellent separation performances.

## 6. Polymer/Oxide Nanoparticles Mixed Matrix Membranes

Oxide nanoparticles, such as silica, alumina, TiO_2_, etc., are often used as filler in MMMs because the particles could modify the properties of neighbor polymers and the interface between the fillers and the polymers, and increase or decrease the path to be traveled by one of the gases, leading to increased permeability [169]. Table 5 summarizes the recent separation performance of oxide nanoparticles-based MMMs. Hosseini et al. prepared an MMM based on a combination of MgO nanoparticles surfaced-modified with Ag^+^ and Matrimid^®^ 5218 polyimide matrix. The surfaces of MgO nanoparticles (NPs) have high affinity towards CO_2_, and surface-anchored Ag^+^ further enhances the solubility of CO_2_ through the formation of reversible π-complexation with double bonds. The obtained Matrimid–MgO–Ag^+^ MMM with 20 wt.% inorganic loading showed 50% and 35% increases in selectivity for CO_2_/CH_4_ and CO_2_/N_2_, respectively, compared to the neat membranes [170]. Ahn et al. reported a preparation of MMM comprising silica NPs and polymer with intrinsic microporosity (PIM). The permeability of the MMM increased with the addition of silica NPs due to the void cavities created by the impermeable particles. However, the selectivity of CO_2_/N_2_ in the membrane decreased due to the addition of the NPs [171]. Ahmad et al. reported an MMM comprising TiO_2_ NPs with an average particle size of 21 nm and a PVAc matrix. With up to 10 wt.% of the TiO_2_ loaded, the membrane showed improvements in both thermal stability and gas permeation. At 2 bar and 30 °C, the permeability of O_2_, CO_2_ and H_2_ was increased by 95%, 79% and 62%, respectively. The selectivities of the gas pairs O_2_/N_2_, H2/N_2_, and CO_2_/N_2_ were also increased by 38%, 26.5% and 14%, respectively [172]. Farashi et al. studied the effect of adding ZnO to the PEBAX matrix. With a 10 wt.% loading of 18 nm ZnO NPs on Pebax-1657, the CO_2_ permeability and CO_2_/CH_4_ selectivity increased about 13 and 21%, respectively [173]. Ameri et al. investigated the effects of adding alumina NPs to polyurethane membranes on gas separation performance. It was found that with the increase in Al_2_O_3_, the permeability of the gases decreased, along with an increase in selectivity. With 30 wt.% of alumina, the selectivity of CO_2_/CH_4_ and CO_2_/N_2_ increased from 7.77 and 25.84 to 23.48 and 67.88, respectively, at 760 mmHg [174]. Molki et al. found similar effects of nickel oxide (NiO) NPs on the permselectivity of polyurethane (PU). When the NiO content was low (1 wt.%), the CO_2_/N_2_ selectivity increased by 79.21%, with a slight increase (~1%) in CO_2_ permeability. However, when the inorganic load increased to 5 wt.%, the CO_2_/N_2_ selectivity increased by 161.1%, while the CO_2_ permeability was reduced by 3.31% [175].

**Figure 10:**
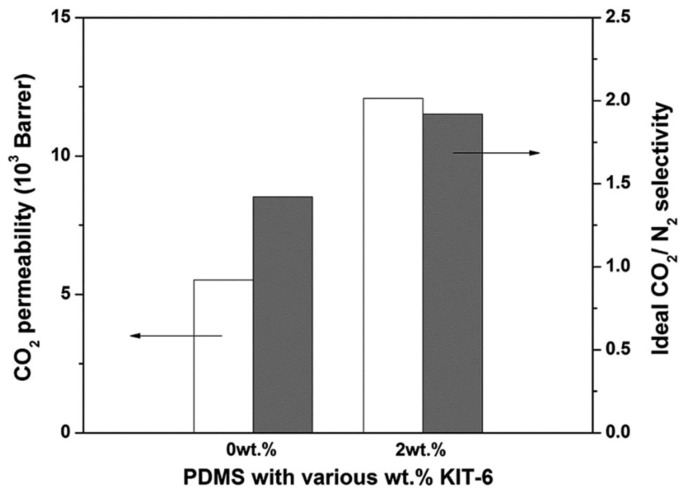
CO_2_ permeability and CO_2_/N_2_ selectivity for 0 and 2 wt.% p-KIT-6/PDMS MMM at 0.5 bar at 35 °C. Reprinted with permission from [183], copyright 2015 RSC.

Mesoporous particles with relatively large pores could be an ideal filler for an MMM due to the extra channel provided for gas diffusion. Wang et al. explored the use of mesoporous KIT-6 silica, which has a larger pore size (~6 nm) and a three-dimensional interconnected cubic pore structure that favors gas diffusion. With an optimum loading of 2 wt.%, the permeability and selectivity of CO_2_/N_2_ simultaneously increased because the condensation of CO_2_ enabled its passing through the otherwise unpassable, partially blocked mesopores (Figure 10). This effect is even stronger with more condensable gases, such as C_4_H_10_, with which the permeability and selectivity against N_2_ increased by 464% and 248% [183].

**Figure 11:**
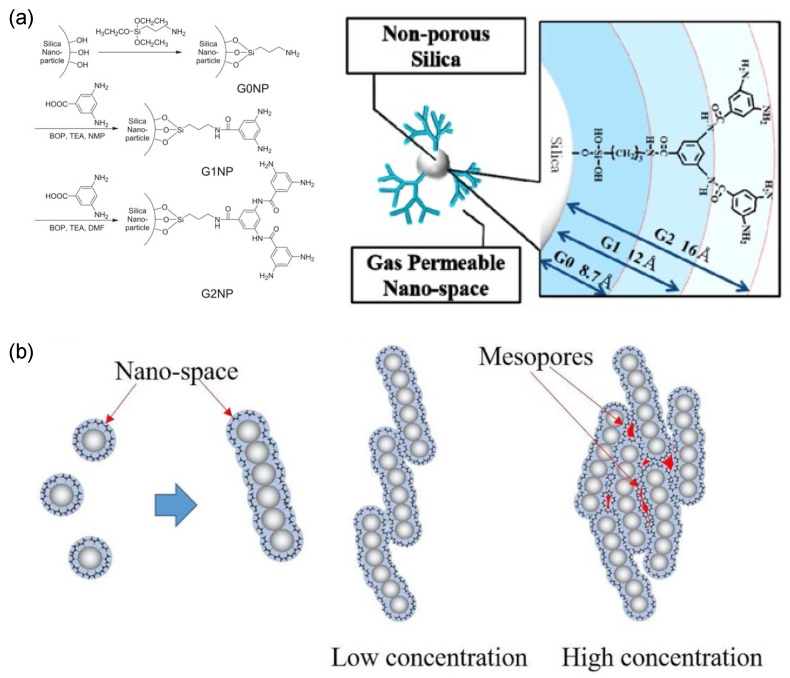
(**a**) Surface modification of silica nanoparticles to generate dendritic amino nanospace. Reprinted with permission from [58], copyright 2017 Elsevier. (**b**) Change from spherical particles to pearl necklace particles for the formation of efficient gas transport channels via the assembly of surface-modified and structure-specific nanoparticles at high concentrations. Reprinted with permission from [177], copyright 2020 Elsevier.

Surface modification of silica nanoparticles by APTES can enhance the permeability without reducing the selectivity. Su et al. reported that in an MMM comprising crosslinked PEG and SiO_2_ NPs, the permeability was increased by 20% to 133 Barrer with 2.7 wt.% inorganic loading [176]. To further increase the permeability of SiO_2_-containing MMMs, Hasebe et al. developed a type of polyimide-based MMM with a permeable nanospace by anchoring dendritic amino groups onto the surfaces of silica nanoparticles (Figure 11). The surface modification prevented particle aggregation, while the dendritic amino groups provided extra nanospace, which enhances the solubility and permeability of CO_2_. An MMM comprising 6FDA-DABA polyimide and 25 wt.% inorganic content showed an improved CO_2_ permeability of 1920 Barrer without significant reductions in CO_2_/N_2_ selectivity (23) [58]. Building on the idea of using NPs with peripheral nanospace, Kudo et al. increased the loading of the silica NPs with nanospace up to 50 wt.%, so that the particles would start to aggregate anisotropically to form aggregations of pearl necklace-like structures. This gave rise to much nanospace, together with newly formed mesopores that can increase permeability without sacrificing selectivity. The MMMs with 50 wt.% dendritic amino silica in PIM showed permeabilities of 152,000, 2850, 916 and 1340 Barrers, respectively for CO_2_, O_2_, N_2_ and CH_4_, and a CO_2_/N_2_ selectivity of 16.6, a CO_2_/CH_4_ selectivity of 11.4, and an O_2_/N_2_ selectivity of 3.1, surpassing the 2008 Robeson upper bound [177].

Polymer-grafted nanoparticles have recently been explored in the context of CO_2_ separation due to their increased mean free volume and higher permeability compared to neat polymers [184]. However, the increase in permeability is often associated with a drastic drop in selectivity. Bilchak et al. recently found that the distribution of free volume is spatially heterogeneous, with larger pores residing on the far-end of the grafted polymer chain. They discovered that by adding long free polymer chains in a polymer-grafted NP matrix, such larger pores can be blocked to prevent the diffusion of larger gas molecules without significantly affecting the permeability of smaller gas molecules. Based on this design, an MMM was prepared comprising 3% long free poly(methyl acrylate) (PMA) chains (molecular weight (MW) = 96,000) and PMA-grafted silica NPs, which are synthesized via surface-initiated reversible addition−fragmentation chain transfer polymerization (SI-RAFT), with a chain length of MW of 100,000 and grafting density of 0.5 chains/nm^2^. Such an MMM showed a CO_2_ permeability of 42.6 Barrer and a CO_2_/CH_4_ selectivity of 23.2 at an equimolar binary feed ratio [178].

Polyhedral oligomeric silsequioxane (POSS) is a silica nanoparticle containing an inorganic silica and oxygen-caged core with organic functional groups attached. The organic functional end groups allow for POSS to be compatible with a wide range of polymers. Therefore, POSS can be used as an organic filler nanocomposite in membrane design due to its excellent mechanical properties, ability for dimensional building blocks, and flexibility for enhanced separation. Various polymer–POSS interactions have been studied for gas separation in CO_2_ capture from methane CH_4_, nitrogen (N_2_) and hydrogen (H_2_) gas [185].

Chemically manipulated POSS nanoparticles with different polymers can help enhance CO_2_ separation in membranes. A recent study examined the CO_2_/CH_4_ separation ability of a pure graphene oxide (GO) membrane. Typically, GO membranes are restricted based on the size and interlayer interactions of the 2D structure. Yang et al. researched the incorporation of amino-functional POSS into GO membrane structures [179]. POSS–NH_2_ has a rigid structure that can regulate the interlayer structure of the GO and overall increase the selectivity while decreasing swelling. The GO–POSS–NH_2_ membrane yielded a CO_2_/CH_4_ selectivity of 74.5, compared to the pure GO membrane, which had a CO_2_/CH_4_ selectivity of 10.

Kim et al. synthesized co-polymer POSS–PEG with a side chain of polymethlhydrosiloxane (PMHS), which was used as the starting polymer and was casted on a polyacrylonitrile (PAN) and polydimethylsiloxane (PDMS) membrane support composite [180]. Increased amounts of allyl-POSS caused the membranes to become brittle due to their high crystallinity, whereas high amounts of allyl-PEG enhanced the flexibility. However, large amounts of PEG resulted in membranes unable to be cast as free-standing. The copolymer PEG–POSS with a ratio of 80:20 resulted in the best CO_2_/N_2_ permeability of 679 Barrer and a selectivity of 38.1. Ahmad et al. reported amino-functionalized POSS with polysulfone (PSf)-soaked ionic liquid (IL) [181]. IL is an excellent CO_2_ adsorbent and has high thermal stability. IL-PSf/POSS membranes displayed an increase in selectivity for CO_2_/N_2_ of about 24.12, and a CO_2_ permeance of 34.98 GPU.

Optimizing the content of POSS can also improve CO_2_/H_2_ separation. Ansaloni et al. fabricated ammonium chloride-functionalized POSS with 6FDA reactive precursors for tubular and disk polyPOSS-imide membranes [182]. Pure and mixed gas tests in N_2_, CH_4_, H_2_ and CO_2_ showed that polyPOSS-imide had a CO_2_/N_2_ selectivity of 18. The permeation of the H_2_ decreased compared with pure gas conditions, potentially due to the concentration polarization in the feed gas. However, Chung et al. showed that Pebax/POSS had the best CO_2_ permeability of 135 Barrer at 8 atm pressure, with na CO_2_/H_2_ selectivity of 52.3 for mixed gas [186]. It was observed that integrating Pebax/POSS into an MMM enabled a better separation performance in mixed gas than pure gas due to CO_2_ plasticization. The plasticization helped increase the free volume and chain mobility.

Polymer–POSS allows for greater tunability and flexibility. Future studies will continue to better our understanding of different polymers with POSS, enabling us to achieve better CO_2_ separation from either N_2_, CH_4_ or H_2._ These polymer/nanocomposites can be incorporated into MMMs and other membrane supports to enhance certain desired properties for CO_2_ gas separation.

Of these reported MMMs, PU-based MMMs and commercial polymer Matrimid-based MMMs gave some of the best selectivities, while PIM-based MMMs achieved some of the best permeability values. POSS-based inorganic fillers tend to require relatively low amounts of loading compared to other oxide-based nanoparticles.

## 7. Polymer/Nano Carbon Mixed Matrix Membranes

In the past three decades, nanocarbon materials have emerged as promising filler materials for making MMMs due to their unique surface functionality, variety of 3D structures, and other superior physical properties. These carbon materials include 0-D carbon quantum dots, 1-D carbon nanotubes (CNT) and carbon fibers, 2-D graphene and graphene oxide (GO), 3-D carbon molecular sieves and activated carbons, etc. Table 6 lists some of the recent examples of polymer/carbon MMMs.

**Figure 12:**
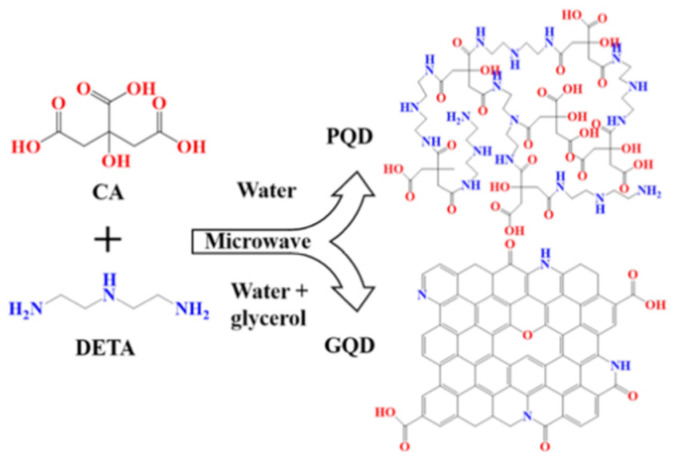
Schematic of the formation of nanocarbon-based QDs. Reprinted with permission from [187], copyright 2020 ACS.

Quantum dots are 0-D nanomaterials of a few nanometers with different properties from larger particles due to quantum mechanics. Shi et al. investigated the effect of adding polymer-like quantum dots (PQD) and graphitic-like QDs (GQD) to a PEBAX matrix on gas separation performance (Figure 12). They found that, while PQD destroyed the crystalline structures of the polymer matrix, GQD with fewer functional groups caused an enhancement in microphase separation of PEBAX with only 0.05 wt.% loading, which led to more segregated domains for selective CO_2_ permeation. As a result, only 0.05 wt.% of GQD loading is needed to achieve an optimal gas separation property, while 1 wt.% is needed for PQD [187].

The 1-D carbon nanotubes achieve transport diffusivity of gases that is orders of magnitudes higher than that of zeolites with comparable pore sizes, possibly due to their atomically smooth inner walls, which lead to less friction for mass transport [199]. Therefore, MMMs based on the use of either single-walled carbon nanotubes (SWCNTs) or multi-walled carbon nanotubes (MWCNT) have been reported. Kim et al. reported MMMs based on dispersing SWCNT-modified long alkyl chains in a PSf polymer matrix. It was found that adding SWCNT significantly increased the permeability, but with a reduced selectivity for CO_2_/CH_4_ [188]. Ahmad et al. prepared β-CD-functionalized MWCNT/cellulose acetate (CA) MMMs via the wet-phase inversion technique. Such MMMs with only 0.1 wt.% filler loading showed improved gas permeability and selectivity for CO_2_/N_2_ [189]. Zhao et al. prepared an MMM comprising MWCNT–NH_2_ in a Pebax 1657 matrix. The incorporation of the MWCNT–NH_2_ increased the amorphous region of Pebax, which was beneficial for gas permeability. With the addition of 33 wt.% of MWCTN, the permeability of CO_2_ increased 3-folds, characterized by a significant decrease in the activation energy of permeation for CO_2_ and no loss of selectivity for CO_2_/N_2_, CO_2_/H_2_ or CO_2_/CH_4_ [190]. Zhang et al. created a hydrogel-based MMM by wrapping MWCNT with a polyNIPAM hydrogel layer within a Pebax 1657 matrix. The MWCNT served as a highway for gas transport, while the hydrogel layer favors both CO_2_ permeability and selectivity. Such MMMs in a humidified state showed a high CO_2_ permeability of 567 Barrer, and selectivities of 35 and 70 for CO_2_/CH_4_ and CO_2_/N_2_ [191].

The 2-D graphene oxides (GO) have also been studied as fillers in MMMs for CO_2_ separation [200]. However, individual GO nanosheets are impermeable to gas and liquids due to their high electron density. As a result, the conventional blending of GO with polymers results in MMMs with relatively low permeability. For example, Ha et al. fabricated PDMS/GO composite elastomer membranes. With 8 wt.% of GO added, the MMM showed a more than 99.9% reduction in gas permeation towards various gases, including H_2_, O_2_, N_2_, CH_4_ and CO_2_, as well as an increase in CO_2_/N_2_ and CO_2_/CH_4_ selectivity over two order of magnitudes greater than neat PDMS [192]. On the other hand, due to the abundance of oxygen-based functional groups and the increased interfacial gaps, with a minor addition of GO nanosheets, the solubility of the CO_2_ can be considerably increased, resulting in higher selectivity as well as higher permeability. Shin et al. added 0.3 wt.% GO nanosheets to a Pebax/PEG matrix, and the MMM showed both improved CO_2_/N_2_ selectivity (55.8) and CO_2_ permeability (~650 Barrer). Moreover, such GO-based MMMs showed outstanding anti-plasticization resistance (up to 9 bar) and long-term stability (~100 days) under mixed gas conditions [193]. Using GO as additives to achieve anti-aging was also demonstrated in MMMs using PIM-1 as a polymer phase, which is a type of membrane material notorious for fast aging. Luque-Alled et al. developed chemically crosslinked PIM-1/GO hybrids MMM by covalently attaching PIM-1 to GO functionalized with APTS (APTS-GO), and they found that with 10 wt.% addition of APTS-GO, the membrane maintains its initial CO_2_ permeability after 150 days, which is 9 times longer than the pure PIM-1 membrane [201].

One way to achieve higher permeability and selectivity is by functionalizing GO nanosheets with amino groups. Zhang et al. modified GO nanosheets with aminosilane to obtain amino-GO/Pebax MMM. The surface modification improved the filler distribution and membrane mechanical strength. More importantly, the surface amino groups constructed a better CO_2_ transport highway. In the dry state, with the addition of 0.5 wt.% of the amino-functionalized GO nanosheet, the CO_2_ permeability increased from 95.4 Barrer to 172 Barrer. The performance is even more impressive under the humid state. With 0.9 wt.% of inorganic filler in the humid state, the MMM showed improved CO_2_ permeability from 424 Barrer to 934.3 Barrer at 2 bar and 35 °C, while the CO_2_/CH_4_ selectivity increased to 40.9 and the CO_2_/N_2_ selectivity increased to 71.1 [194].

**Figure 13:**
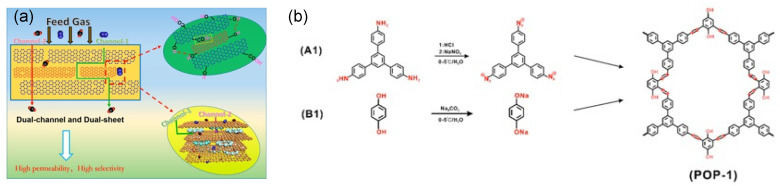
(**a**) Schematic illustration of design of POP-PGO-Pebax MMMs. (**b**) Chemical structure and synthesis procedure of o-POP. Reprinted with permission from [196], copyright 2019 ACS.

Another way to improve the permeability of GO-based MMMs is by using porous reduced GO (PRG). Dong et al. prepared PRG-based MMMs based on a Pebax 1657 matrix with a finely tuned degree of GO reduction to ensure sufficient residual amounts of functional groups on the reduced GO. The narrow gas flow galleries with an average gap of 0.34 between neighboring reduced GO sheets ensured a high molecular sieving effect, while the generated mesopores on the laminate produced rapid gas transport pathways. As a result, the MMM showed substantially improved CO_2_ permeability (119) as well as CO_2_/N_2_ selectivity (104) at 0.2 MPa and 30 °C [195]. In another work, He et al. reported a layer-by-layer CO_2_-philic Pebax MMM that was functionalized with both porous graphene oxide (PGO) and a “N_2_-phobic, CO_2_-philic” o-hydroxyazo-hierarchical porous organic polymer (o-POPs) (Figure 13). Such MMMs exhibited a high CO_2_ permeability of 232.7 Barrer, with an ideal selectivity of CO_2_/N_2_ of 80.7 [196].

CNT and GO can be used simultaneously in an MMM to give both high permeability and selectivity by taking advantage of the smooth inner wall of CNT and the molecular sieving effect of GO. Li et al. prepared a Matrimid polyimide-based MMM with 5 wt.% MWCNT and 5 wt.% GO. Compared to the neat membrane, such MMMs showed an increase in CO_2_ permeability, CO_2_/CH_4_ selectivity and CO_2_/N_2_ selectivity of 331%, 149% and 147% respectively [197].

3-D carbon materials, such as micron-sized activated carbon (CA) particles, have also been explored as effective fillers in MMMs. Anson et al. created MMMs by adding micro-mesoporous activated carbon (AC) particles, with a particle size of 0.9 micron and surface area of 3271 m^2^/g, into an acrylonitrile–butadiene–styrene (ABC) polymer matrix. At 20 °C, with 10 wt.% of the AC, the MMM showed an increase in CO_2_ permeability from 2.87 to 10.81 Barrer, with a CO_2_/CH_4_ selectivity increase from ~24 to 35. Alternatively, by adding 40 wt.% of AC with a particle size of 4.47 micron and surface area of 818 m^2^/g, the MMM achieved a CO_2_ permeability of 20.5 with a CO_2_/CH_4_ selectivity of 51 at 20 °C [198].

In these nanocarbon-based MMMs, the commercial polymers Pebax and Matrimid are among the most widely adopted polymer matrix. GO-based membranes require the lowest level of loading, and have a major impact on the improvement of selectivity. On the other hand, CNT-based MMMs tend to lead to huge improvement in terms of permeability.

## 8. Conclusions

In this mini review, we have examined recent advances in mixed matrix membranes based on five major types of fillers: zeolite, MOF, ZIF, oxide nanoparticles and nanocarbons. While attentions have been focused on single-gas permeabilities and ideal gas selectivities, it is worth noting that studies examining MMMs under practical conditions are extremely important. Moreover, mechanical strength, resistance to aging and plasticization, and cost/easiness of fabrication are equally important for developing practically viable membranes. While most MMMs are based on non-covalent blending, MMMs based on covalent grafting have shown unique advantages, especially given that thermodynamic phase separation could occur in non-covalent systems. Moreover, current MMMs are based on an inorganic-in-polymer design, but with the advances in membrane fabricating techniques and polymer chemistries, polymer-in-inorganic MMM could be possible, which would achieve both high permselectivities beyond the Robeson upper bound, and good mechanical integrity.

Because of the promise of MMMs, many companies, such as NuMat Technologies, have started the application of such materials in commercial business cases. However, several barriers need to be overcome before commercial success can be realized. For example, most research papers are performed under lab conditions, and more aggressive gas testing is needed to meet industry conditions, focusing on long-term durability, high pressure, and the presence of impurity gases. Additionally, as the fabrication process starts to be scaled up, aggregations tend to occur during membrane preparation, especially for membranes with high inorganic loadings. In addition, the fabrication cost and materials prices of potential MMMs need to be further reduced in order to compete with pure polymer alternatives.

## Figures and Tables

**Table 1:** Transport properties of zeolite/polymer mixed matrix membranes.

Zeolite	Polymers	Pressure/Temp	Zeolite Loading	CO_2_ (Barrer)	CH_4_ (Barrer)	N_2_ (Barrer)	H_2_ (Barrer)	CO_2_/CH_4_	CO_2_/N_2_	H_2_/CO_2_	Ref.
Zeolite 13X	PEBAX	14 Bar/25 °C	1 wt.%	194.1				56	56.5		[65]
Zeolite T	6FDA-durene	3.5 Bar/30 °C		843.6	44.2			19.1			[66]
Zeolite 4A	PVAc	0.1 MPa/30 °C	25 wt.%	2.413		0.024	3.75		100.54		[67]
Li/Na-SZM-25	Matrimid^®^ 5218	5 bar/35 °C	5 wt.%	12				169			[69]
Zeolite NaY	PEBAX/PEG	0.15 MPa/35 °C	30 wt.%	172.6		1.6			107.9		[70]
Hierarchical Zeolite 5A	Carbonized Matrimid^®^ 5218	1 bar/35 °C	30 wt.%	2450				19.3			[71]
Zeolite 4A	PVAc	440 psi/35 °C	50 vol%	11.4	0.457				25.0		[68]
Aminosilanized Zeolite 13X	6FDA-Durene	0.2 MPa/RT	15 wt.%	887					25.3		[72]
Aminosilanized Zeolite EMC-2	6FDA-ODA	150 psig/35 °C	15 wt.%	40.9	0.51			80.2			[73]
Aminosilanized NaY zeolite	Matrimid^®^ 5218	2 Bar/35 °C	15 wt.%	9.7				57.1			[74]
Aminosilanized zeolite 3A	PSf	12 Bar/25 °C	40 wt.%	4.22 GPU			15.1 GPU			7.12	[75]
Aminosilanized MCM-41	PSf	10 Bar/25 °C	30 wt.%	9.13				31.48	32.97		[76]
SAPO-34 with ionic liquid	PSf	3.5 Bar/30 °C	5 wt.%	7.19 GPU				44.9			[77]
ZSM-5 with ionic liquid	6FDA-TeMPD	75 mmHg/35 °C	15 wt.%	142	9.48	5.55	32.6	15.0	25.6		[78]
SAPO-34	PIL-RTIL	2 atm/25 °C	25 wt.%	260				90			[79]
SAPO-34	PIL-RTIL	40 bar/17 °C	30 wt.%	202				43 (binary feeds)			[80]

**Table 2:** Transport properties of MOF/polymer mixed matrix membranes.

MOFs	Polymers	Pressure/Temp	MOF Loading %	CO_2_ (Barrer)	CH_4_ (Barrer)	N_2_ (Barrer)	H_2_ (Barrer)	CO_2_/CH_4_	CO_2_/N_2_	CO_2_/H_2_	Ref.
UiO-66-NH_2_	PSf	3 bar/35 °C	30	46				~24–26			[88]
UiO-66-CN	PIM-1	1 bar/25 °C	20	12,063.3		225.5			53.5		[89]
UiO-66-NH_2_	PVP_PEI	1 atm/25 °C	18	394						12	[90]
PA-UiO-66	PVA	>1.3 ka/40 °C	0.5							20.3	[91]
UiO-66-NH_2_	Pebax-1657	2–15 bar/25 °C	50	325 GPU				20	56		[92]
NH_2_–MIL-53(Al)	Pebax	10 bar/35 °C	10	149				23.3	59.4		[93]
NH_2_–MIL-53(Al)	CA	3 bar/25 °C	15					16	12		[94]
ZIF-8	6FDA-BI	4 bar/35 °C	20	20.3	0.35	0.78	78.5	58	25.9		
ZIF-8	Zn^2+^(0.01 g/mL)	4 bar/35 °C	20	15.9	0.23		72.3	70.2	26.7		[95]
ZIF-8	PS-co-SBC	2 bar/25 °C	20	40					22		[96]
MKP	PVAm/mPSf	0.5 MPa °C	44.4	164.6					242		[97]
Y-fum-fcu-MO	6FDA-DAM	3.5 bar/−40–56 °C	20	<1000				130			[98]
UiO-66-NH_2_ @PI	ODPA-DAM	3.1 bar/35 °C	17	103				51	24		[99]
UiO-66-NH_2_ @PI	ODPA-DAM	3.1 bar/35 °C	27	142				43	27		[99]
66-NH_2_@Ni-MOF-74 shell	ODPA-DAM	3 bar/35 °C	22	90				44			[100]

**Table 3:** Transport properties of unmodified ZIF/polymer mixed matrix membranes.

ZIFs	Polymers	Pressure/Temp	ZIF Loading	CO_2_ (Barrer)	CH_4_ (Barrer)	N_2_ (Barrer)	H_2_ (Barrer)	CO_2_/CH_4_	CO_2_/N_2_	H_2_/CH_4_	Ref.
ZIF-8	6FDA-Durene	3.5 bar/30 °C	10 wt.%	1426	49.71			28.7			[133]
ZIF-8	P84 polyimide	3 bar/25 °C	17 wt.%	6.33	0.068			93.6			[136]
ZIF-8	Pebax 1657	11 bar/35 °C	2 wt.%	118	5.5	2		21.4	59		[130]
ZIF-8	PVC-g-POEM	1 bar/35 °C	26.8 wt.%	224	15.7	5.6		14.3	40		[132]
ZIF-8	PSF	4 bar/30 °C	0.5 wt.%	3.25	0.21	0.19		15.1	17.5		[126]
ZIF-8	Pebax 1657	0.5 MPa °C	5 wt.%	140					67		[125]
ZIF-11	Pebax 2533	2 bar/20 °C	50 wt.%	230	20	5		12	47		[137]
ZIF-11	6FDA-DAM	4 bar/30 °C	20 wt.%	257	8.3		272	31		32	[127]
ZIF-67	Pebax 1657	11 bar/35 °C	4 wt.%	16	5.8	2.2		27.6	72.7		[130]
ZIF-71	6FDA-Durene	3.5 bar/35 °C	20 wt.%	2560	181	186		14.2	13.8		[134]
ZIF-90	6FDA-DAM	4.5 bar/35 °C	15 wt.%	720				37			[138]
ZIF-90	Triptycene-PI	9.8 atm/35 °C	10 wt.%	26	0.6	1.1	61	42	24	99	[123]
ZIF-95	Matrimid	4 bar/35 °C	30 wt.%	23.2	0.4		76.6	58		192	[135]
ZIF-301	6FDA-DAM	4bar/25°C	20 wt.%	891				29.3			[124]

**Table 4:** Transport properties of modified and/or hybrid ZIF-based mixed matrix membranes.

ZIFs and Hybrids	Polymers	Pressure/Temp	ZIF Loading	CO_2_ (Barrer)	CH_4_ (Barrer)	N_2_ (Barrer)	H_2_ (Barrer)	CO_2_/CH_4_	CO_2_/N_2_	H_2_/CH_4_	Ref.
Modified ZIFs											
OH-ZIF-7	Pebax 2533	4.5 bar/20 °C	14 wt.%	273					38		[156]
ZIF-8@PD	6FDA-TBDA22	1 bar/35 °C	7 wt.%	380	15	20	600	25	19	40	[143]
ZIF-8@PEG200	Matrimid 5218	8 bar/25 °C	30 wt.%	33.1	2.4			15.4			[160]
Amino-ZIF-7	PEG	5 bar/35 °C	36 wt.%	215				55			[142]
Amino-ZIF-8	Pebax 1657	0.1 MPa/25 °C	6 wt.%	164					62		[158]
Amined-ZIF-8	6FDA-Durene	3.5 bar/25 °C	1 wt.%	550	32.4			17			[154]
Amined-ZIF-8	PSF	4 bar/30 °C	0.5 wt.%	4.2	0.19	0.15		22.3	27.8		[126]
NH2-ZIF-8	Polyamine TNF	1 Mpa/25 °C	0.5 wt.%	211GPU					51		[141]
ZIF-8@VR	PAA	1.5 bar/35 °C	66.8 wt.%		1410						[155]
ZIF-8@IL	Pebax 1657	1 bar/25 °C	15 wt.%	104.9				34.8	83.9		[151]
ZIF-8@Ni	Pebax 2533	2 bar/25 °C	10 wt.%	321					42.8		[140]
ZIF-8@metakaolin	PVAm	1 bar/25 °C	3 wt.%	169 GPU					86.7		[144]
ZIF-8@Zn(II)	6FDA-BI	4 bar/35 °C	20 wt.%	20.3	0.35	0.78	78.5	57.9	25.9	223	[95]
Hybrid ZIFs											
ZIF-301/CNTs	PSF	2 bar/25 °C	18 wt.%	19					48		[146]
ZIF-302/CNTs	PSF	2 bar/25 °C	18 wt.%	18					35		[148]
ZIF-300/CNTs	Ultrason	2 bar/25 °C	30 wt.%	19.9					50.8		[145]
ZIF-302/GO	PSF	1 bar/25 °C	18 wt.%	11.5	0.25				45.9		[147]
ZIF-8/GO	Matrimid	1 bar/30 °C	20 wt.%	238					65		[153]
ZIF-8/GO	PI-ODPA-TMPDA	1 bar/25 °C	10 wt.%	142				41			[150]
ZIF-8/GO	Pebax 2533	6 bar/25 °C	6 wt.%	249					47.6		[157]
ZIF-8/GO	XLPEG	1 bar/25 °C	6 wt.%	475					58.2		[159]
ZIF-8/MIL-101 (Cr)	PSF	200 kpa/35 °C	16 wt.%	14					40		[152]
ZIF-8/MWCN	Pebax 1657	0.5 Mpa/35 °C	8 wt.%	186					61.2		[149]
ZIF-8/SiO2	PSF	4 bar/30 °C	0.5 wt.%	3.6	0.23	0.21		15	16		[126]

**Table 5:** Transport properties of unmodified oxide nanoparticle/polymer mixed matrix membranes.

Nanoparticles	Polymers	Pressure/Temp	NP Loading	CO_2_ (Barrer)	CH_4_ (Barrer)	N_2_ (Barrer)	H_2_ (Barrer)	CO_2_/CH_4_	CO_2_/N_2_	H_2_/CO_2_	Ref.
MgO-Ag^+^	Matrimid^®^ 5218	3.5 bar/35C°	20 wt.%	4.31	0.102	0.155	22.7	42.3		5.26	[170]
SiO_2_	PIM	4 psig/23 °C	6.7 vol%	6200		460	3670		15		[171]
TiO2	PVAc	2 bar/30 °C	10 wt.%	5.26		0.07	9.20		74.3		[172]
ZnO	PEBAX	2 bar/30 °C	10 wt.%	149.81	6.26			23.9			[173]
Al_2_O_3_	PU	76 cmHg/35 °C	20 wt.%	74.67	3.18	1.10		23.48	67.89		[174]
NiO	PU	1 bar/30 °C	5 wt.%	321	14.75	4.74		21.76	67.72		[175]
Amino SiO_2_	XLPEG	NA/35 °C	2.7 wt.%	134	6.4	2.1		22	62		[176]
Dendritic Amino SiO_2_	6FDA-DABA	76 cmHg/35 °C	25 wt.%	1920				23			[58]
Dendritic Amino SiO_2_	PIM-1	76 cmHg/35 °C	50 wt.%	15,200	1340	916		11.4	16.6		[177]
PMA-g-SiO_2_	PMA	24 bar/35 °C Equimolar feed	~20 wt.%	42.6	1.84			23.2			[178]
Amino POSS	GO	2 bar/25 °C	5 wt.%					74.5			[179]
PEG-POSS	PMHS	2 bar/25 °C	80 wt.%	679				38.1			[180]
IL-POSS	PSf	3.5 bar/25 °C	5 wt.%	34.98 GPU		1.45 GPU			24.1		[181]
POSS	6FDA polyimide	10 bar/35 °C	~50 wt.%	424 GPU			2547 GPU			6.3	[182]

**Table 6:** Transport properties of nanocarbon/polymer mixed matrix membranes.

Nanoparticles	Polymers	Pressure/Temp	NP Loading	CO_2_ (Barrer)	CH_4_ (Barrer)	N_2_ (Barrer)	H_2_ (Barrer)	CO_2_/CH_4_	CO_2_/N_2_	H_2_/CH_4_	Ref.
Carbon QD	Pebax	1 bar/25 °C	0.05 wt.%	668					101		[187]
Alkyl SWCNT	PSf	4 bar/35 °C	5 wt.%	5.12	0.27	0.23		18.82			[188]
β-CD-MWCNT	CA	3 × 10^5^ Pa/35 °C	0.1 wt.%	741.67		18.46			40.17		[189]
NH_2_-NWCNT	Pebax	0.7 MPa/35 °C	33 wt.%	361				16	52		[190]
PNIPAM-NWCNT	Pebax	2 atm/25 °C (Humid state)	5 wt.%	567				35	70		[191]
GO nanosheet	PDMS	8 wt.%	10 Bar/35 °C	27.7					24		[192]
GO nanosheet	Pebax/PEG	1 bar/35 °C	0.3 wt.%	~650					55.8		[193]
Amino-GO nanosheet	Pebax	2 bar/35 °C (Humid state)	0.9 wt.%	934.3				40.9	71.1		[194]
Porous reduced GO	Pebax	0.2 MPa/30 °C	5 wt.%	119					104		[195]
oPOP-porous GO	Pebax	1 atm/25 °C	2 wt.%	232.7					80.7		[196]
MWCNT+GO	Matrimid	0.2 MPa/30 °C	5 wt.% each	38.07	0.45	0.47		84.6	81.0		[197]
Activated carbon	ABS	0.2 MPa/20 °C	40 wt.%	20.5				51			[198]

## Data Availability

Not applicable.
